# Anabolic role of lysyl oxidase like-2 in cartilage of knee and temporomandibular joints with osteoarthritis

**DOI:** 10.1186/s13075-017-1388-8

**Published:** 2017-08-02

**Authors:** Weam Alshenibr, Mustafa M. Tashkandi, Saqer F. Alsaqer, Yazeed Alkheriji, Amelia Wise, Sadanand Fulzele, Pushkar Mehra, Mary B. Goldring, Louis C. Gerstenfeld, Manish V. Bais

**Affiliations:** 1 Department of Molecular and Cell Biology, Boston University Henry M. Goldman School of Dental Medicine, W-216, 700 Albany Street, Boston, MA 02118 USA; 2 Department of Orthopaedic Surgery, School of Medicine, Boston University, Boston, MA 02118 USA; 3 Department of Orthopaedic Surgery and Institute of Regenerative and Reparative Medicine, Georgia Regents University, Augusta, GA 30912 USA; 4 Department of Oral and Maxillofacial Surgery, Boston University Henry M. Goldman School of Dental Medicine, 100 East Newton Street, Boston, MA 02118 USA; 5 Hospital for Special Surgery Research Institute, Weill Cornell Medical College, New York, NY 10021 USA; 6 Department of Cell and Developmental Biology, Weill Cornell Medical College, New York, NY 10021 USA

**Keywords:** LOXL2, Anabolic response, Cartilage, Osteoarthritis

## Abstract

**Background:**

Lysyl oxidase like-2 (LOXL2) is a copper-dependent amine oxidase. Our previous studies showed that LOXL2 is elevated during mouse fracture healing. The goal of this study was to evaluate the potential of LOXL2 to act as an anabolic agent in cartilage affected by osteoarthritis (OA).

**Methods:**

LOXL2 was visualized in tissues from human knee and hip joints and temporomandibular joints (TMJ) by immunofluorescence. The activity of LOXL2 in human articular and TMJ chondrocytes was assessed by cell-based assays, microarray analysis, and RT-qPCR, and LOXL2-mediated activation of NF-κB and extracellular signal-related kinase (ERK) signaling pathways was measured by western blotting. To examine LOXL2-induced effect in vivo, we implanted Matrigel-imbedded human chondrocytes into nude mice and exposed them to exogenous LOXL2 for 6 weeks. Finally, LOXL2-induced effects on collagen type 2 α1 (COL2A1) and phospho-SMAD2/3 were evaluated by immunofluorescence analysis.

**Results:**

LOXL2 staining was detected in damaged regions of human TMJ, hip and knee joints affected by OA. Stimulation with transforming growth factor (TGF)-β1 upregulated LOXL2 expression, while pro-inflammatory cytokines IL-1β and TNF-α downregulated LOXL2, in human chondrocytes. Viral transduction of LOXL2 in OA chondrocytes increased the mRNA levels of chondroitin sulfate proteoglycan (CSPG4), aggrecan (ACAN), sex determining region Y-box containing gene 9 (SOX9), and COL2A1 but reduced the levels of extracellular matrix (ECM)-degrading enzymes matrix metalloproteinase (MMP)1, MMP3, and MMP13. Further, forced expression of LOXL2 promoted chondrogenic lineage-specific gene expression, increased the expression of *COL2A1* in the presence of TNF-α, and inhibited chondrocyte apoptosis. LOXL2 expression also inhibited IL-1β-induced phospho-NF-κB/p65 and TGF-β1-induced ERK1/2 phosphorylation. Matrigel constructs of human chondrocytes from the knee joint and TMJ implanted in nude mice showed anabolic responses after LOXL2 transduction, including increased expression of *SOX9*, *ACAN*, and *COL2A1*. Finally, immunofluorescence staining revealed co-localization of LOXL2 with SOX9 in the nuclei of cells in the implants, decreased phospho-SMAD2/3, and increased COL2A1 staining.

**Conclusion:**

Our results suggest that although LOXL2 is upregulated in cartilage affected by OA, this may be a protective response that promotes anabolism while inhibiting specific catabolic responses in the pathophysiology of OA.

**Electronic supplementary material:**

The online version of this article (doi:10.1186/s13075-017-1388-8) contains supplementary material, which is available to authorized users.

## Background

Osteoarthritis (OA), the most common degenerative joint disease, leads to structural damage and ultimately loss of function and disability. Progressive, irreversible destruction of the joints in OA is driven by defective cartilage extracellular matrix (ECM) remodeling and the loss of chondrocytes due to apoptosis [1]. Knee pain and symptomatic knee OA have increased in prevalence [2], and similar manifestations of the disease process are seen in temporomandibular joint (TMJ) disorders [3]. Advances in understanding the disease pathogenesis are critical to OA prevention and treatment.

Lysyl oxidase (LOX) family members (LOX, LOXL1-4) are copper-dependent amine oxidases that function in ECM remodeling and collagen cross-linking. LOX activity is required for the formation of immature and mature pyrodinoline (PYR) cross-links in native and engineered cartilage. Hypoxia-induced LOX increases PYR cross-links and tensile properties of the articular cartilage, knee meniscus, patellar tendon, and anterior and posterior cruciate ligaments, while exogenously applied LOX proteins are capable of enhancing collagen cross-linking and cartilage tissue functional properties [4]. Our previous studies showed that a member of the LOX family, LOXL2, is elevated during fracture healing in mice and that it promotes chondrogenesis and the formation of cartilage ECM [5, 6]. Whether LOXL2 plays any role in the pathophysiology of OA and the effect on cartilage is not known.

Pro-inflammatory cytokines such as tumor necrosis factor (TNF)-α and interleukin (IL)-1β are catabolic cytokines [7] involved in chondrocyte apoptosis and matrix proteolysis; they contribute to the pathophysiology of OA [8]. Anti-cytokine agents have been evaluated for OA therapy, but with limited success [9, 10]. Anabolic factors that have roles in chondrogenesis and articular cartilage maintenance have been used as additive factors in tissue engineering strategies and are considered as candidate structure-modifying agents for OA therapy [11]. Examples are fibroblast growth factor-18 [12] and members of the bone morphogenetic protein 2 (BMP)/transforming growth factor β1 (TGF-β) family. Differential chondrocyte responses due to aging and OA may account for off-target effects in joints treated with pro-anabolic agents; such effects can result in cartilage calcification, osteophyte formation, angiogenesis, and synovial fibrosis [13, 14]. Thus, alternative approaches to regulating these inflammatory cytokines could lead to improved therapies.

To better understand the role of LOXL2 in OA cartilage, we used global gene expression, immunohistochemical, and mouse xenotransplantation studies to assess the effects of LOXL2 overexpression in OA chondrocytes in vitro and in vivo. Our findings indicate that LOXL2 may act as a pro-anabolic agent in OA cartilage, providing a foundation for future work on OA therapy.

## Methods

### Human tissues and animal experiments

Human tissues were obtained with Institutional Review Board (IRB) approval and informed consent from all subjects. TMJ tissues were obtained from Boston University (IRB H33300). TMJ tissues were selected on the basis of clinical features such as TMJ pain and mobility and confirmed by pathological diagnostic findings as healthy or TMJ-OA, at the Department of Pathology, Boston University. The exclusion criteria included a history of any rheumatic disease or cancer. Histological slides from 16 patients (8/condition) including TMJ normal (Osteoarthritis Research Society International (OARSI) grade I) and TMJ-OA (OARSI grade V) from right and left joints were obtained. Freshly isolated cells from TMJ-OA cartilage from five patients were used for animal experiments. Histological slides of tissues from hip joints were obtained from the Department of Orthopaedic Surgery, School of Medicine, Boston University, Boston, MA, USA (IRB H-32517). Tissue sections included both uninvolved and involved regions of the femoral head based on micro computed tomography (microCT) mapping of the regions of cartilage erosion relative to bone cysts. Histological sample slides were obtained from six individuals. The OA cartilage histological tissue slides from the knee joints were obtained from Georgia Medical College, Augusta, GA, USA under IRB approval (657441-5). Tibial epiphyses from OA-affected regions (OARSI grade V) and the adjacent healthy region (OARSI grade I) were collected from five patients. All mouse experiments were performed with guidance and regulations through Boston University Institutional Animal Care and Use Committee (IACUC), approval number AN-15387.

### Immunofluorescence of human tissue sections

Human tissues were fixed in 4% paraformaldehyde overnight, paraffin-embedded, and sectioned for subsequent immunofluorescence by standard procedures. The tissue sections were exposed to anti-LOXL2-specific antibodies (GeneTex) or isotype control antibody; primary antibodies were detected with anti-biotin antibody combined with streptavidin-conjugated Texas red. Anti-fade reagent with 4′,6-diamidino-2-phenylindole (DAPI) was added to all the samples. Sections were viewed under epifluorescence microscopy (Zeiss 710). Quantification of images was performed using Image J software (NIH), as shown in an earlier study [15]. Briefly, the region of interest was selected by drawing the margins. Parameters were set to measure area, integrated density, and mean fluorescence for stained regions, and data were obtained from several regions. Next, the same measurements were obtained from adjacent regions as a background control. We calculated the total corrected cellular fluorescence (TCCF):

$$ \mathrm{TCCF} = \mathrm{Integrated}\ \mathrm{density}\ \hbox{--}\ \left(\mathrm{Area}\ \mathrm{of}\ \mathrm{selected}\ \mathrm{cell} \times \mathrm{Mean}\ \mathrm{fluorescence}\ \mathrm{of}\ \mathrm{background}\ \mathrm{readings}\right) $$

Data are reported as the fold-change expression compared to respective controls.

### RNA isolation and analysis

Total RNA was extracted from paraffin-embedded sections of the femur, tibia, and TMJ condyle using Trizol (Qiagen) and FFPE RNA isolation kit (Qiagen), according to the manufacturer’s instructions. Briefly, tissue sections were deparaffinized with Xylenes for 10 min, washed with Xylenes then ethanol, and air-dried. Tissues were treated with proteinase K and RNAase inhibitor overnight and extracted with Trizol. Finally, RNA was precipitated using 10 μg glycogen and isopropanol, washed with ethanol, and reconstituted in nuclease-free water. Quantitative real-time PCR (RT-qPCR) analysis was performed using TaqMan gene expression assays from Life Technologies, according to a standard protocol [16]. TaqMan® Gene Expression Assay IDs for human and mouse primers were: Sex determining region Y-box containing gene 9 (SOX9) (Hs01001343_g1 and Mm00448840_m1), matrix metalloproteinase (MMP)13 (Hs00233992_m1 and Mm00439491_m1), aggrecan (ACAN) (Hs00153936_m1 and Mm00545794_m1), collagen type II α1 (COL2A1) (Hs00264051_m1 and Mm01309565_m1), and LOXL2 (Hs00158757_m1).

### Human cell cultures

Human articular chondrocytes (HAC) from three OA patients (HAC-OA) (Cell Application Inc. from lot no. 2356, 1777, and 2881) were used. Normal HAC (HAC-N) cells were from Lonza (CC-2550). Cell lines were grown in chondrocyte growth medium (CGM™ BulletKit™, CC-3216, Lonza Inc.) or in chondrocyte differentiation medium (CDM BulletKit, CC-3225, Lonza), both of which contain 5% fetal bovine serum (FBS), R3-insulin-like growth factor-1, human recombinant fibroblast growth factor-beta; insulin-transferrin-selenium (ITS), and gentamycin/amphotericin-B for in vitro studies. The chondrocyte differentiation medium also contains TGF-β3. These supplements have been optimized for HAC [17, 18]. The medium, with a similar composition, from Lonza and Cell Applications optimized for HAC (HAC-O and HAC) has been used in several in vitro and in vivo studies [19, 20] and in our experiments. Primary TMJ cells were obtained as outgrowths from explants of TMJ condylar cartilage within 24 h after collection as 1-mm to 3-mm punch biopsies, and cultured in chondrocyte growth medium with 5% FBS (Lonza Inc.).

### Preparation of lentivirus and adenovirus

Adenoviruses for transient expression of LOXL2 (Ad-CMV-RFP-CMV-hLOXL2-His; referred to as Ad-RFP-LOXL2), and its empty vector (EV) control (Ad-RFP-EV) were custom-synthesized (ADV-214438) by Vector Biolabs. These adenovirus particles were amplified in 293 T cells and quantified using an adenovirus quantification kit (Cell Biolabs). The lentivirus packaging was performed using the 3-plasmid protocol [16]. Lentiviral vector for LOXL2 (CMV-LOXL2) and CMV-EV)) were purchased from Genecopoeia Inc. (EX-Y2020-Lv128 and LV128;). The expression of LOXL2 protein was evaluated by western blotting of the medium and cell layer, and enzymatic activity of purified LOXL2 was evaluated by the Amplex Red assay [21].

### Cell proliferation

The CyQuant cell proliferation reagent assay (Molecular Probes), was used to measure the cell proliferation. HAC or HAC-OA cells were seeded at a density of 20,000 cells per well in 24-well plates and transduced with Adv-RFP-EV or Adv-RFP-LOXL2 in the respective group. After 24 h, they were washed with PBS, and the plates with cell layer were stored at −80 °C. For measurements, plates were thawed at room temperature for 30 min and 200 μl of CyQuant GR dye/cell-lysis buffer added to each well. Fluroscence measured as per manufacturer’s instructions with excitation at 420 nm and emission detection at 535 nm.

### Apoptosis assay

HAC-N or HAC-OA were infected with the Ad-RFP-EV or ad-RFP-LOXL2 at a multiplicity of infection (MOI) of 50 in triplicate wells. The next day, cells were treated with 10 ng/mL of IL-1β, 10 ng/mL of TNF-α, or vehicle for 24 h in serum-free medium. Cells were lysed using lysis buffer provided in the kit and were analyzed with a bioluminescence-based caspase-3/7 glow apoptosis assay (Promega) according to manufacturer’s instructions.

### Molecular signaling analysis

HAC-OA, transduced with either Ad-RFP-LOXL2 or Ad-RFP-EV at MOI 25, were incubated in serum-free growth medium for at least 16 h and then left untreated (vehicle) or treated for 10 min with 10 ng/mL of IL-1β or TGF-β1. Proteins were extracted for western blot analysis.

### Western blotting

Tissue or cell layers were extracted with SDS-PAGE sample buffer (0.1 mM Tris-HCl, 4% SDS, 10% glycerol, 5% β-mercaptoethanol) and boiled for 3–5 minutes. Protein concentrations were determined using Nano Orange assay kits (Molecular Probes, Eugene, OR, USA). From tumors, approximately 20 μg of protein per experimental group were then subjected to 10% SDS-PAGE and western blotting with primary antibodies (Cell Signaling Technology). The antibodies used were for evaluation of phospho-NF-κB-p65 (S536) and NF-κB-p65 (total), phospho-ERK1/2, total extracellular signal-related kinase (ERK) and β-actin. Anti-rabbit and anti-mouse secondary antibodies were purchased from Cell Signaling Technology. Quantification was performed using a digital densitometry system (Versadoc; BioRad, Hercules, CA, USA) and Image J software.

### Gene array experiments

To evaluate the genes that were differentially regulated by LOXL2, HAC-OA from three different OA patients were cultured in chondrocyte growth medium (Lonza) until they reached confluence and were infected with Ad-RFP-EV or Ad-RFP-LOXL2 at MOI of 25 in medium supplemented with 5% FBS. After overnight incubation, cells were washed once with PBS and cultured overnight in the fresh serum-free medium. Cells were processed for total RNA extraction using the RNeasy kit (Qiagen). The sample integrity was verified using RNA 6000 Nano Assay RNA chips run in an Agilent 2100 Bioanalyzer (Agilent Technologies). Analysis of mRNA was performed on microarrays and by TaqMan RT-qPCR. All microarray procedures were performed at the Boston University Microarray Resource Facility, according to the protocol for the GeneChip® WT Plus Kit Reagent Manual (Affymetrix,). The total RNA (100 ng) was reverse-transcribed using GeneChip® WT Plus Reagent Kit (Affymetrix,). The cDNA was used as a template for in vitro transcription to antisense cRNA, which was purified using Purification Beads (Affymetrix,) and then used as a template for reverse transcription to produce single-stranded DNA in the sense orientation. Microarrays were immediately scanned using Affymetrix Gene Array Scanner 3000 7G Plus (Affymetrix). Log2 (expression) was computed across all samples and laid over a colored representation (heatmap), which was scaled so that red and blue indicated expression values ≥2 standard deviations above and below, respectively, the row-wise mean (white).

### Gene array analysis

Affymetrix GeneChip Human Gene 2.0 ST CEL files were normalized to produce gene-level expression values using the implementation of the Robust Multiarray Average (RMA) [22] in the Affymetrix package (version 1.36.1) [23] included in the Bioconductor software suite (version 2.12) [24] and an Entrez gene-specific probeset mapping (version 17.0.0) from the Molecular and Behavioral Neuroscience Institute (Brainarray) at the University of Michigan [25]. Array quality was assessed by computing relative log expression (RLE) and normalized unscaled standard error (NUSE) using the affyPLM Bioconductor package (version 1.34.0). Principal component analysis (PCA) was performed using the “prcomp R” function with expression values that had been normalized across all samples to a mean of zero and a standard deviation of one. Pairwise differential expression was assessed using the moderated (empirical Bayesian) *t* test implemented in the limma package (version 3.14.4) (i.e., creating simple linear models with lmFit, followed by empirical Bayesian adjustment with eBayes). Fold changes are indicated as 2 = twofold higher or −2 = twofold lower in Ad-RFP-LOXL2- than in Ad-RFP-EV-transduced cells. Correction for multiple hypothesis testing was accomplished using the Benjamini-Hochberg false discovery rate (FDR) and represented as FDR q values. All statistical analyses were performed using the R environment for statistical computing (version 2.15.1).

### In vivo implantation of human articular and TMJ chondrocytes in nude mice

As an alternative to various models based on human chondrocytes [26–28], we established a model using implants of primary TMJ-OA or HAC-OA chondrocytes embedded in Matrigel. For in vivo applications, freshly isolated TMJ-OA cells or frozen cells obtained from Cell Application Inc., were expanded only once to maintain their phenotype prior to preparation of implants. Freshly prepared 10^6^ TMJ-OA or HAC-OA chondrocytes in 50 μL medium, from TMJ or knee joints of three different patients with OA, were mixed with Matrigel at a 1:1 ratio. The total 100 μL of Matrigel:chondrocyte suspension were implanted subcutaneously in the backs of nude mice (three implants/mouse) and allowed to grow for a week. These implants were treated locally with weekly injections of 30-μL suspensions of Ad-RFP-LOXL2 or Ad-RFP-EV (n = 5/condition), for 6 weeks. Transduction was confirmed by visualization of RFP by in vitro imaging systems (IVIS) each week. One implant from each mouse was then processed for RNA isolation, and the other two were prepared for histologic analysis and stained with Safranin O/Fast Green (American Mastertek Inc.).

### Immunofluorescence analysis of human cartilage implants extracted from mice

Paraffin-embedded tissue sections were labeled with mouse SOX9 antibody (Abcam) or its isotype control (Vector Biolabs) detected with anti-mouse IgG conjugated to Alexa 488. To detect LOXL2 expression, the tissues were subjected to rabbit anti-LOXL2 (GeneTex), anti-RFP (Abcam), anti-phospho-SMAD2/3 (Cell Signaling Technology), anti-COL2A1 (Abcam) or its isotype control antibody and detected with anti-rabbit Alexa 488-conjugated antibody or biotin antibody followed by streptavidin-conjugated Texas red, in respective samples. Anti-fade reagent with DAPI was added to all samples. A Zeiss 710 dual scanner confocal microscope with a Plan-Apochromat objective, oil immersion lens, and a CCD detector was used to obtain confocal images. Image acquisition was performed with Zeiss Zen image analysis software (Carl Zeiss Micro Imaging). The image analysis was performed using Zeiss LSM viewer and Image J software (NIH). Z-stack images analysis and 3-dimensional reconstruction was performed by using LOCI and the 3D viewer plug-in of Image J software. Quantification was performed using Image J software as described [15].

### Data analysis

Data analyses were performed using two-way analysis of variance (ANOVA) with Bonferroni post-hoc analysis or Student’s *t* test (Graph Pad Prism 5 software). All experiments were performed three times each using cells derived from a different HAC-N, HAC-OA, or TMJ patient sample. Each data point is represented in the graphs as mean ± SEM of three experiments with significance set at *P* < 0.05.

## Results

### Immunolocalization of LOXL2 protein in human TMJ and hip and knee joints

**Fig. 1:**
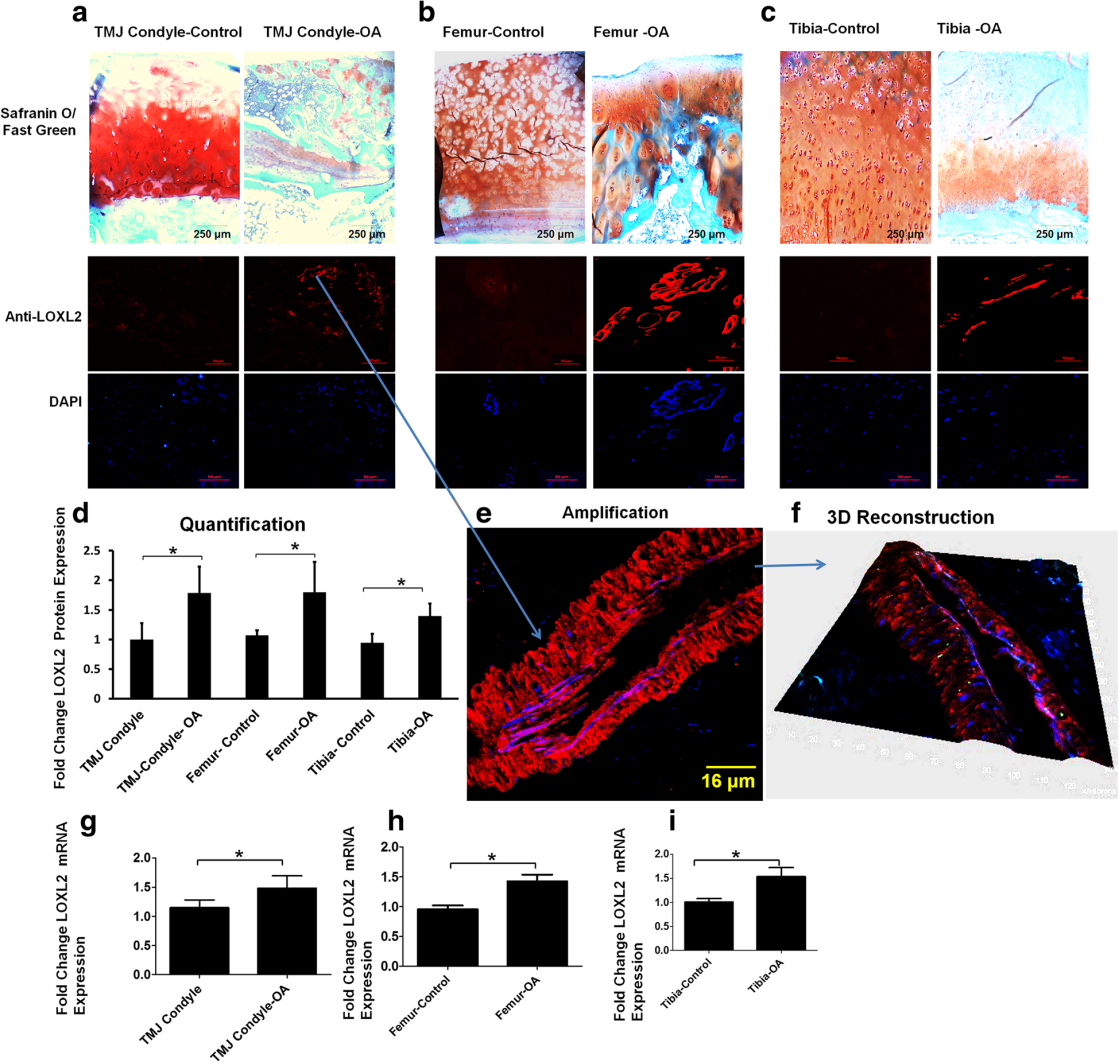
Immunolocalization of LOXL2 protein in the human temporomandibular joints (*TMJ*) and hip and knee joints: Safranin O/Fast green staining of cartilage (*orange*/*green*, *upper panels*), immunofluorescence staining with LOXL2 antibody (*red*, *middle panels*), and 4′,6-diamidino-2-phenylindole (*DAPI*) staining (*blue*, *lower panels*) in human TMJ condyles from normal and TMJ-osteoarthritis (*TMJ-OA*) patients **a** and unaffected and OA-affected regions of the femoral head from the same patient (**b**), unaffected and OA-affected regions of the tibial plateau from the same patient (**c**), with quantification of staining intensities in OA tissues (fold-change) compared to normal/unaffected (set at 1.0) (**d**). **e** Higher magnification of a damaged region from a TMJ-OA specimen, where LOXL2 is expressed. **f** Three-dimensional reconstruction of the LOXL2-expressing region. RT-qPCR analysis of LOXL2 mRNA in a normal TMJ condyle compared to a TMJ-OA condyle (**g**), unaffected control compared to hip OA (femoral head) (**h**), and unaffected control compared to knee OA (tibial plateau) (**i**). *Scale bar* = 250, 50, or 16 μm as indicated in *panel*; n = 5/condition; *P < 0.001 (Student’s *t* test)

To determine whether LOXL2 is present in OA tissues, we evaluated its localization by immunohistochemistry in healthy (OARSI, grade I) and OA tissues (OARSI grade V) from multiple joints, including the TMJ condyle, the femoral head, and the tibial plateau of the knee joint. Safranin O/Fast green staining was used to identify the cartilage region (Fig. 1a). LOXL2 was detectable in damaged parts of cartilage, compared to the negligible LOXL2 in the healthy regions of tissues from all three joints (Fig. 1). Thus, LOXL2 is upregulated in damaged joints, similar to other anabolic factors such as BMP2 [29], fibroblast growth factor (FGF)18 [30], insulin-like growth factor (IGF)1, and TGF-β1 [31], compared to cartilage not affected by OA.

### Regulation of LOXL2 mRNA by OA-related factors

**Fig. 2:**
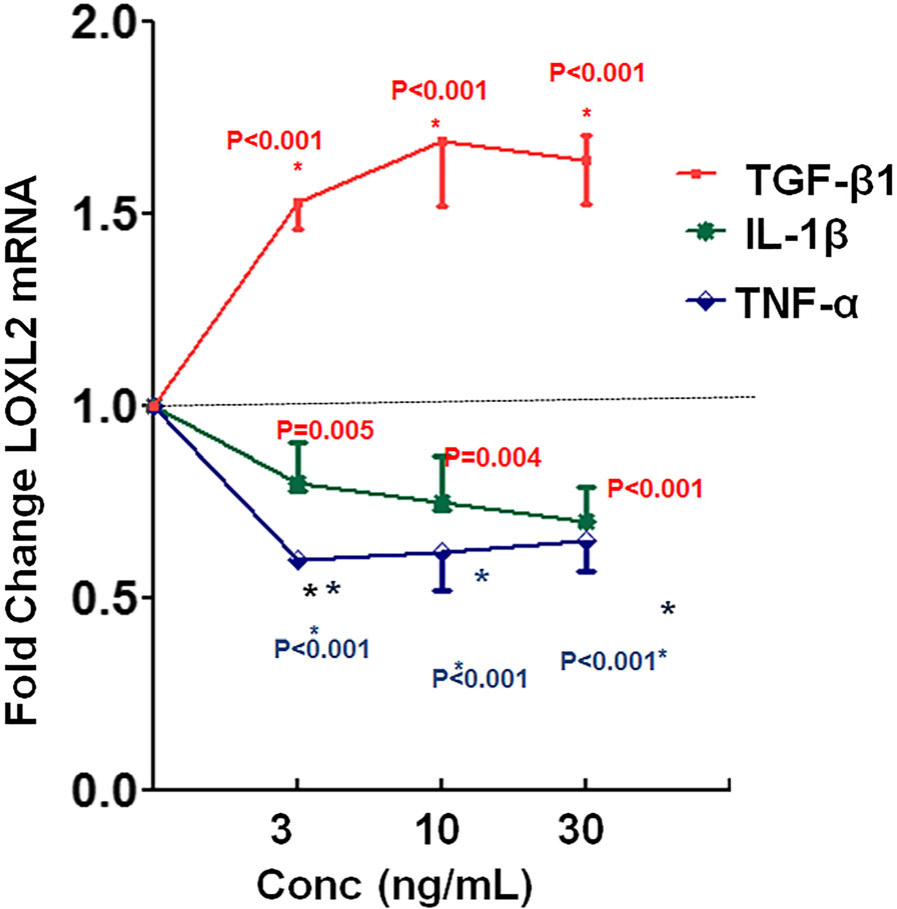
Regulation of LOXL2 gene expression by osteoarthritis (OA)-related mediators: Human articular OA chondrocytes (HAC-OA) were treated with TNF-α, IL-1β, or transforming growth factor-β1 (*TGF-β1*) at 10 or 30 ng/mL for 24 h, and LOXL2 mRNA levels were assessed by RTqPCR. Data are represented as mean ± SEM of three independent experiments with 95% confidence interval (CI) (*P < 0.001; Student’s *t* test). LOXL2 gene expression was significantly upregulated by TGF-β1 (*P* < 0.001) and downregulated by TNF-α (*P* < 0.001) and IL-1β (*P* < 0.001).

Whether LOXL2 expression is modulated by anabolic and catabolic factors involved in OA [7] has not been defined. Therefore, we investigated the effects of anabolic factors (TGF-β1, IGF-1, BMP-2, and BMP-7) and catabolic factors (TNF-α, and IL-1β) on LOXL2 gene expression in chondrocytes treated for 24 h. Compared to vehicle stimulation, LOXL2 mRNA levels in HAC-OA were induced significantly by TGF-β1 (twofold) stimulation, whereas they were downregulated 0.5-fold and 0.7-fold, respectively, by TNF-α and IL-1β (Fig. 2). However, treatment with IGF-1, BMP-2, or BMP-7 produced no significant effect on LOXL2 mRNA levels in HAC-OA (not shown). These results indicate that LOXL2 gene expression can be modulated by key factors affecting OA-related processes in cartilage.

### LOXL2 promotes the differentiated chondrocyte phenotype and inhibits apoptosis in human chondrocytes from OA cartilage

**Fig. 3:**
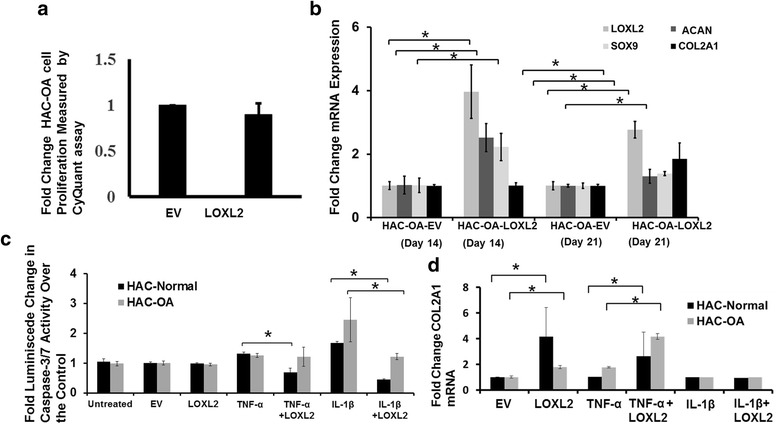
LOXL2 promotes a differentiated phenotype and inhibits apoptosis in human articular chondrocytes from cartilage affected by osteoarthritis (*OA*). **a** Human articular OA chondrocytes (*HAC-OA*) transduced with adenoviruses for transient expression of LOXL2 (Ad-RFP-LOXL2) compared to cells transduced with its empty vector (*EV*) control (Ad-FP-EV) did not affect chondrocyte proliferation after 24 h. **b** Lentiviral cytomegalovirus (CMV)-LOXL2 transduction of HAC-OA compared to CMV-EV increased the mRNA levels of collagen type II α1 (*COL2A1*) (*P* = 0.043), sex determining region Y-box containing gene 9 (*SOX9*) (*P < 0.001), and aggrecan (*ACAN*) (*P* = 0.002). **c** Ad-RFP-LOXL2 transduction inhibited caspase 3/7 activity induced by TNF-α or IL-1β (*P = 0.006) compared to Ad-RFP-EV in HAC-normal (*HAC-N*) and HAC-OA cells in serum-free medium after 24 h. **d** Adenoviral transduction (Ad-RFP-LOXL2) HAC-OA in serum-free medium increased COL2A1 mRNA levels (*P < 0.001) compared to control (Ad-RFP-EV) in the presence of TNF-α (*P = 0.001)

To evaluate if LOXL2 promotes the differentiated chondrocyte phenotype, we examined proliferation, chondrocyte phenotypic markers, and TNF-α-induced and IL-1β-induced effects on COL2A1 mRNA and apoptosis using chondrocytes isolated from healthy cartilage (HAC-N) or OA cartilage (HAC-OA) and transduced with viral vectors. HAC-OA cells infected with Ad-RFP-LOXL2 and cultured for 24 h showed no significant difference in cell proliferation compared to those transduced with Ad-RFP-EV (Fig. 3a). To permit long-term expression of the transduced LOXL2 in the HAC-OA cultures for evaluation of chondrogenic differentiation, we used lentiviral vectors lenti-CMV-LOXL2 and lenti-CMV-EV (control). HAC-OA-LOXL2 cells showed increased SOX9, ACAN, and COL2A1 mRNA levels on days 14 and 21 after lenti-CMV-LOXL2 transduction and culture in chondrogenic differentiation medium compared to cells transduced with lenti-CMV-EV vector (Fig. 3b). Next, we determined that LOXL2 transduction inhibits IL-1β-induced caspase 3/7 activity as a measure of apoptosis in both HAC-N and HAC-OA cells (Fig. 3c). However, LOXL2 transduction inhibited caspase 3/7 activity induced by TNF-α in HAC-N but not in HAC-OA cells. Finally, transduction with Ad-RFP-LOXL2, compared to Ad-RFP-EV, increased COL2A1 mRNA levels in both healthy HAC-N and HAC-OA cells treated with TNF-α for 24 h (Fig. 3d). Thus, exogenous expression of LOXL2 does not affect chondrocyte proliferation, but promotes chondrogenic lineage-specific gene expression, increases *COL2A1* expression in the presence of TNF-α, and inhibits chondrocyte apoptosis.

### LOXL2 overexpression differentially regulates genes in OA chondrocytes

**Fig. 4:**
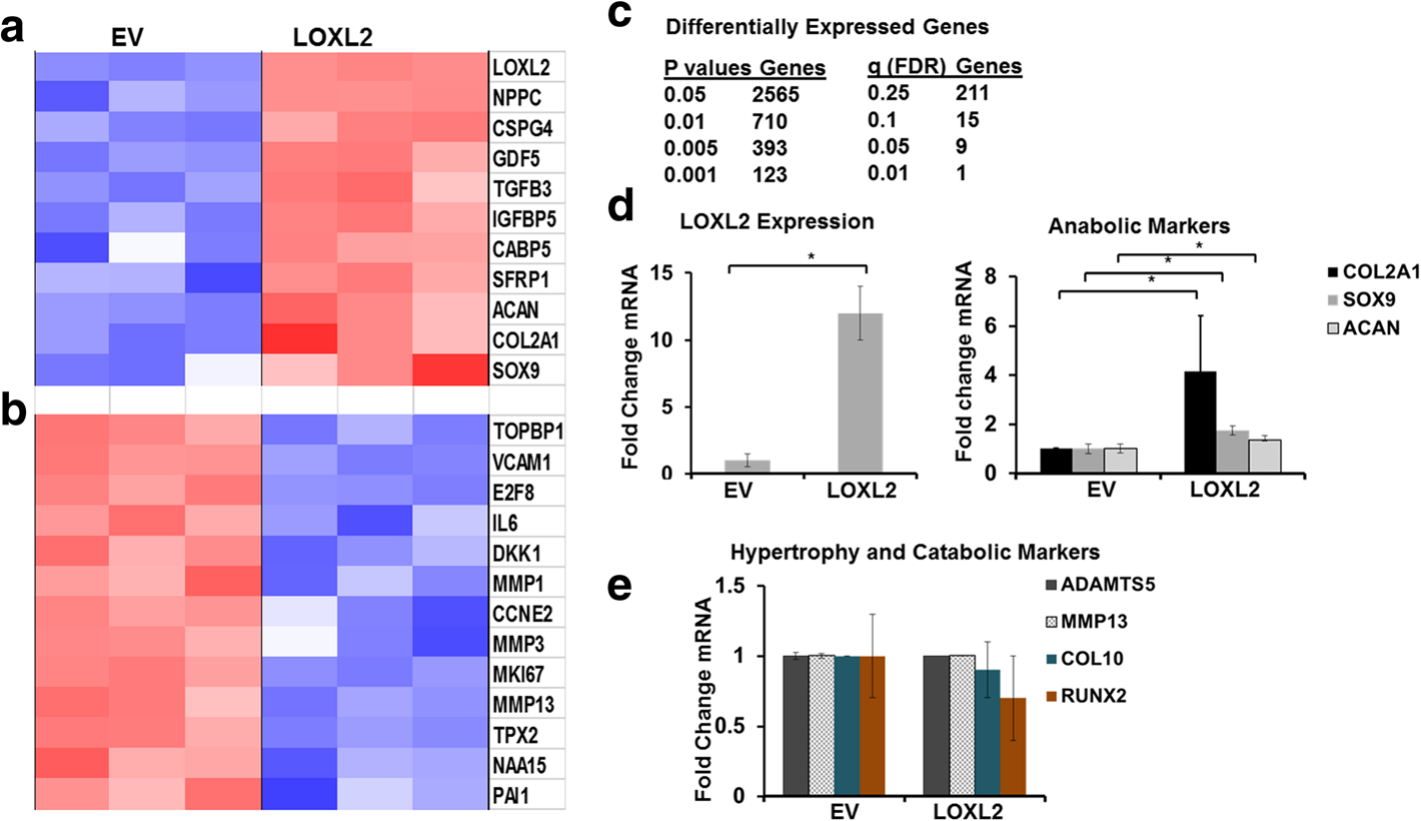
LOXL2 overexpression differentially regulates genes in osteoarthritis (OA) chondrocytes. Human articular chondrocytes from OA cartilage (HAC-OA) were transduced with adenoviruses for transient expression of LOXL2 (Ad-RFP-LOXL2) or Ad-RFP-empty vector (*EV*), and total RNA extracts were subjected to global gene expression analysis. LOXL2 overexpression in HAC-OA induced the upregulated gene signature (**a**) and the downregulated gene signature (**b**). **c** Statistical significance for differential gene expression. **d** Exogenous LOXL2 expression increased anabolic markers in HAC-OA chondrocytes, whereas genes related to chondrocyte hypertrophy and catabolic markers were not changed as validated by RT-qPCR (**e**) (*P < 0.001; Student’s *t* test). LOXL2 transduction increased the expression of collagen type II α1 (*COL2A1*) (*P* = 0.063), Sex determining region Y-box containing gene 9 (*SOX9*) (*P* = 0.002), and aggrecan (*ACAN*) (*P* = 0.003). *NPPC* natriuretic peptide C, *CSPG* chondroitin sulfate proteoglycan, *GDF5* growth differentiation factor 5, *TGFB3* transforming growth factor B3, *IGFBP3* insulin-like growth factor binding protein, *CABP5* calcium binding protein 5, *SFRP1* secreted frizzled-related protein 1, *TOBP1* topoisomerase II binding protein 1, *VCAM1* vascular cell adhesion molecule 1, *E2F8* E2F transcription factor 8, *DKK1* Dickkopf WNT signaling inhibitor 1, *CCNE2* cyclin E2, *TPX* microtubule-associated homolog, *NAA* N(alpha)-acetyltransferase, *FDR* false discovery rate, *MMP* matrix metalloproteinase

To determine the consequences of LOXL2 overexpression on potential OA-related targets, we performed global gene expression analysis on total RNA extracted from HAC-OA after transduction with Ad-RFP-LOXL2 or Ad-RFP-EV. LOXL2 overexpression resulted in the upregulation of genes such as natriuretic peptide C (*NPPC*), *CSPG4*, growth differentiation factor 5 (*GDF5*), *TGFB3*, insulin-like growth factor binding protein 5 (*IGFBP5*), calcium binding protein 5 (*CABP5*), secreted frizzled-related protein 1 (*SFRP1*), *ACAN*, *COL2A1*, and *SOX9* (Fig. 4a, arranged by decreasing FDR value). Exogenous LOXL2 expression reduced the expression of genes (Fig. 4b) such as topoisomerase (DNA) II binding protein 1 (*TOPBP1*), vascular cell adhesion molecule 1 (*VCAM1*), E2F transcription factor 8 (*E2F8*), *IL6*, Dickkopf WNT signaling inhibitor 1 (*DKK1*), matrix metalloproteinase (*MMP*) 1, cyclin E2 (*CCNE2*), *MMP3*, antigen identified by monoclonal antibody Ki-67 (*Ki67*), *MMP13*, microtubule-associated homolog (*TPX2*), and N(alpha)-acetyltransferase (NAA)15. These expression changes were statistically significant (Fig. 4c). Validation of the global gene expression data by RT-qPCR analysis (Fig. 4d) showed that the key genes related to anabolic responses such as *COL2A1*, *SOX9*, and *ACAN* were increased after LOXL2 transduction, whereas those related to catabolic responses and chondrocyte hypertrophy such as ADAMTS5, MMP13, COL10, and RUNX2 were unchanged (Fig. 4e). Thus, our gene array analysis suggests that LOXL2 may be involved in promoting chondrogenic lineage maintenance, acting as an anabolic factor that also inhibits catabolic and angiogenic factors.

### LOXL2 regulates specific gene sets

**Fig. 5:**
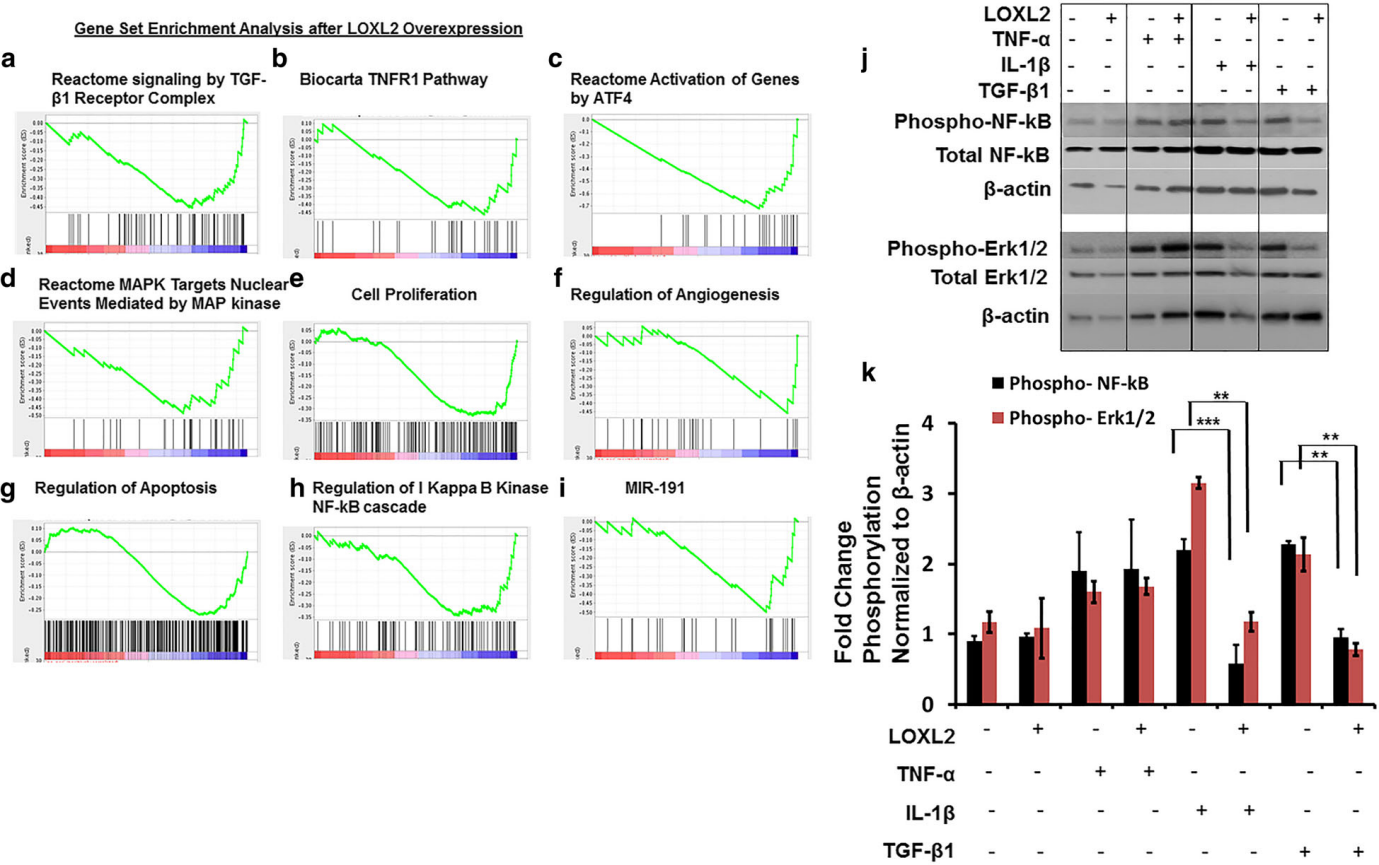
LOXL2 overexpression differentially regulates signaling pathways in osteoarthritis chondrocytes. Gene set enrichment analysis data shown are reactome signaling by transforming growth factor -β1 (*TGF-β1*) receptor complex (**a**), Biocarta TNF-α receptor 1 pathway (**b**), reactome activation genes by ATF4 (**c**), reactome mitogen-activated protein kinase (*MAPK*) targets nuclear events mediated by MAPK (**d**), negative regulation of cell proliferation (**e**), regulation of angiogenesis (**f**), regulation of apoptosis (**g**), regulation of IkB kinase NF-ĸB cascade (**h**), and signaling by MiR-191 **i** compared to control (EV). **j** LOXL2 inhibits IL-1β-induced phospho-NF-ĸB and TGF-β1-induced phospho-ERK1/2 shown by western blot analysis, whereas TNF-α induced phospho-NF-ĸB and phospho-ERK1/2 are not affected. **k** Quantification is shown as fold-change normalized to β-actin (*P < 0.001; Student’s *t* test; n = 3)

In HAC-OA transduced with Ad-RFP-LOXL2 and Ad-RFP-EV, 43 (*P* value <0.01) and 106 (*P* value <0.05) gene sets were enriched significantly; only 3 gene sets were enriched significantly at FDR <25% (0.25) (Table 1). LOXL2 transduction compared to the EV control downregulated genes known to be involved in TGF-β1 and TNF-α signaling and apoptosis (Fig. 5a-i); these include reactome signaling by TGF-β1 receptor complex, Biocarta TNF-α receptor 1 pathway, reactome activation genes by ATF4, negative regulation of cell proliferation, regulation of angiogenesis regulation of apoptosis, reactome mitogen-activated protein (MAP) kinase nuclear events mediated by MAP kinases, regulation of IkB kinase NF-ĸB cascade, and signaling by miR-191.

**Table 1:** GSEA of selected pathways on the basis of OA-related functions from chondrocytes transduced with ADV-LOXL2 compared to empty vector (control)

Name	Size	NES	Nom *P* value	FDR q value
Reactome cell cycle checkpoints	109	−2.478	0.0001	0.00
Reactome activation of genes by ATF4	24	−2.234	0.000	0.00
Reactome SMAD2 SMAD3 SMAD4 heterotrimer regulates transcription	25	−1.880	0.002	0.01
Reactome signaling by TGF Beta receptor complex	59	−1.748	0.0001	0.03
Reactome MAPK targets nuclear events mediated by MAP kinases	30	−1.620	0.010	0.07
ttccgtt, MIR-191	29	−1.604	0.012	0.07
Cell proliferation GO 0008283	495	−1.578	0.000	0.08
Biocarta TNFR1 pathway	29	−1.533	0.033	0.11
Negative regulation of cell proliferation	153	−1.508	0.002	0.12
aagcact, MIR-520 F	238	−1.493	0.000	0.13
Regulation of angiogenesis	26	−1.476	0.061	0.14
Reactome muscle contraction	46	2.023	0.0001	0.19
Regulation of apoptosis	332	−1.373	0.006	0.21
Regulation of cell proliferation	298	−1.371	0.010	0.21
IĸB kinase NF-ĸB cascade	109	−1.36	0.06	0.23
Vasculature development	55	−1.346	0.072	0.24
Reactome platelet calcium homeostasis	16	1.768	0.001	0.24
Reactome antigen activates b cell receptor leading to the generation of second messengers	29	1.750	0.001	0.24
Negative regulation of developmental process	189	−1.335	0.022	0.25

A positive NES indicates that the genes in the set are predominantly upregulated with respect to the reference group, and a negative NES indicates that the genes in the set are predominantly downregulated with respect to the reference group. *GSEA* gene set enrichment analysis, *OA* osteoarthritis, *FDR* false discovery rate, *MAP* mitogen-activated protein, TGF transforming growth factor

### LOXL2 inhibits signaling pathways induced by IL-1β and TGF-β1

We next validated the inhibitory effects of LOXL2 on signaling pathways detected by gene set enrichment analysis (GSEA) (Fig. 5a-i), focusing on those involving NF-κB and ERK. Transduction of HAC-OA with Ad-RFP-LOXL2 inhibited IL-1β-induced phospho-NF-κB/p65 and TGF-β1-induced phospho-ERK1/2 levels compared to cells transduced with Ad-RFP-EV, as shown by western blotting (Fig. 5j and k).

### LOXL2 promotes anabolic responses in implants of the human knee and TMJ-OA chondrocytes in vivo and co-expresses with SOX9

**Fig. 6:**
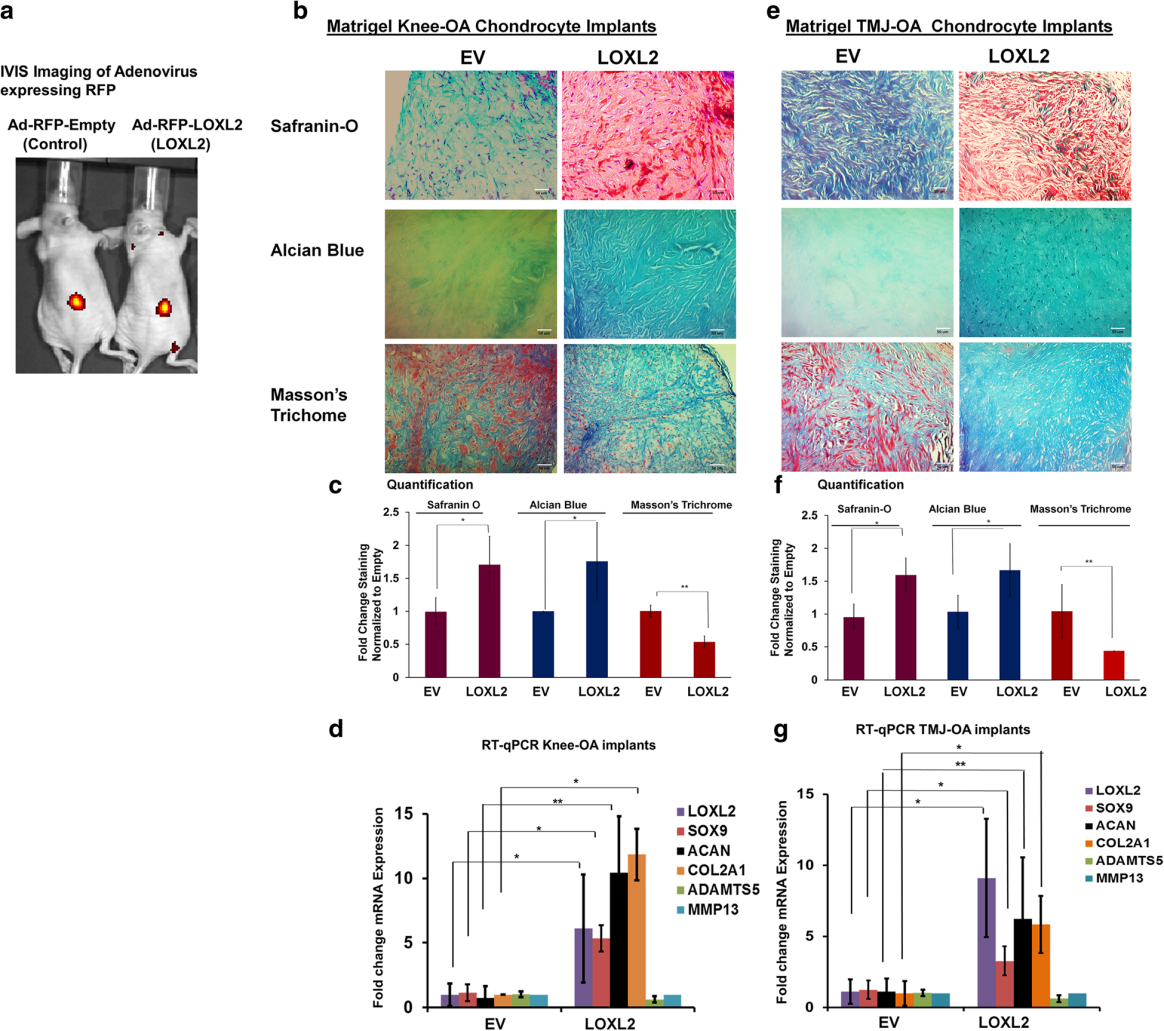
LOXL2 promotes anabolic responses in implants of the human knee and osteoarthritic temporomandibular joint (*TMJ-OA*) chondrocytes in vivo. **a** In vitro imaging systems (*IVIS*) imaging of human articular chondrocytes (HAC)/Matrigel implants in nude mice after local injection every week for 6 weeks with adenoviruses for transient expression of LOXL2 (*Ad-RFP-LOXL2*) or Ad-RFP-empty vector (*EV*) (*Ad-RFP-empty*). Knee HAC-OA/Matrigel implants were injected weekly with Ad-RFP-LOXL2 or Ad-RFP-EV, harvested after 6 weeks, and analyzed by Safranin O/Fast Green, Alcian blue, and Masson’s trichrome staining (**b**) with relative quantification (**c**), and RT-qPCR using human primers (**d**). TMJ-OA/Matrigel implants were injected weekly with Ad-RFP-LOXL2 or Ad-RFP-EV, harvested after 6 weeks, and analyzed by Safranin O/Fast Green, Alcian blue, and Masson’s Trichrome staining (**e**) with relative quantification (**f**), and RT-qPCR (**g**) using human primers (n = 5/condition; *P < 0.05 and **P < 0.01; Student’s *t* test). *SOX*9 sex determining region Y-box containing gene 9, *ACAN* aggrecan, *COL2A1* collagen type II α1, *MMP* matrix metalloproteinase

**Fig. 7:**
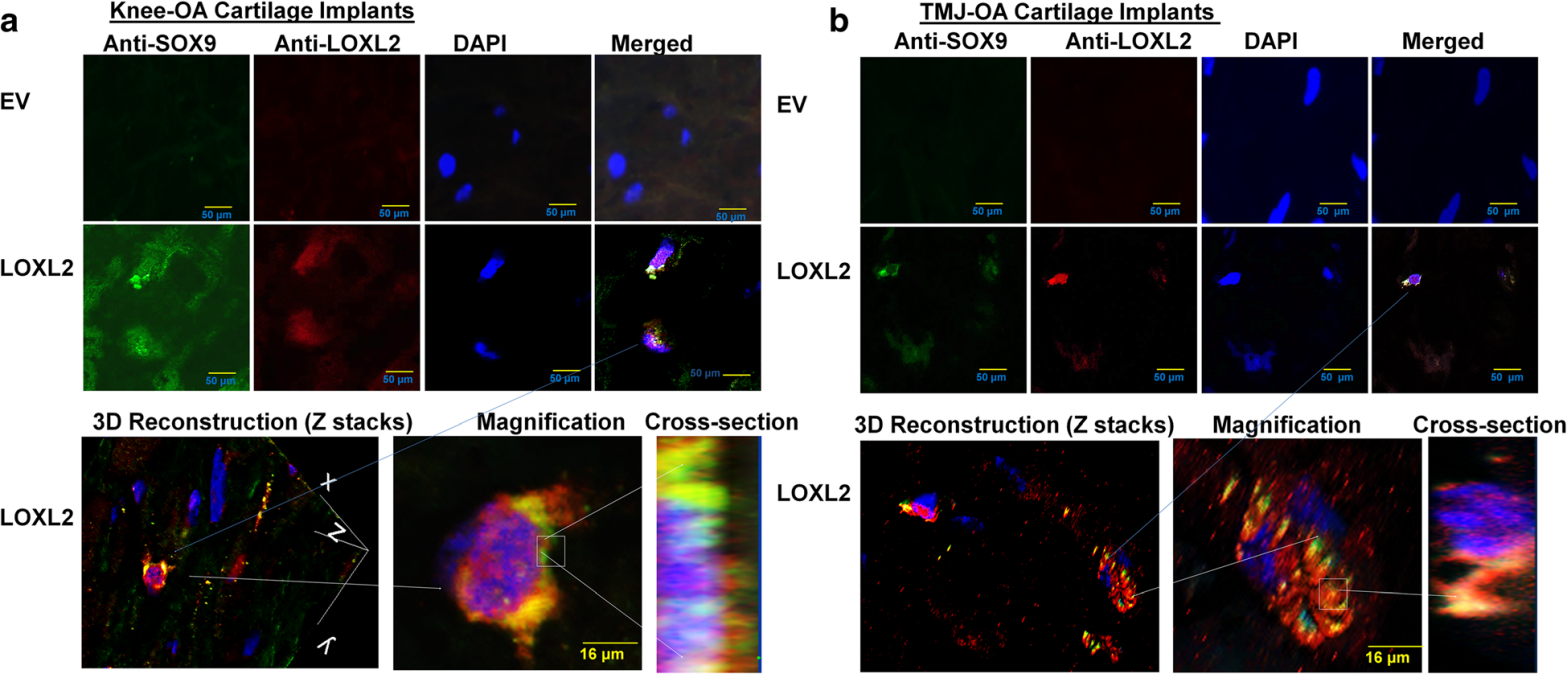
LOXL2 co-expresses with sex determining region Y-box containing gene 9 (*SOX9*) in the knee joint and osteoarthritic temporomandibular joint (*TMJ-OA*) chondrocytes implants in nude mice. **a** Human knee articular chondrocytes in osteoarthritis (*HAC-OA*)/Matrigel implants or TMJ-OA/implants (**b**) from nude mice after treatments as described in Fig. 6. In the *image*, localization of proteins is shown with different *colors* such as LOXL2 (*red*), 4′,6-diamidino-2-phenylindole (*DAP*I) (*blue*), and LOXL2 and SOX9 co-localization (*yellow*). *EV* empty vector

**Fig. 8:**
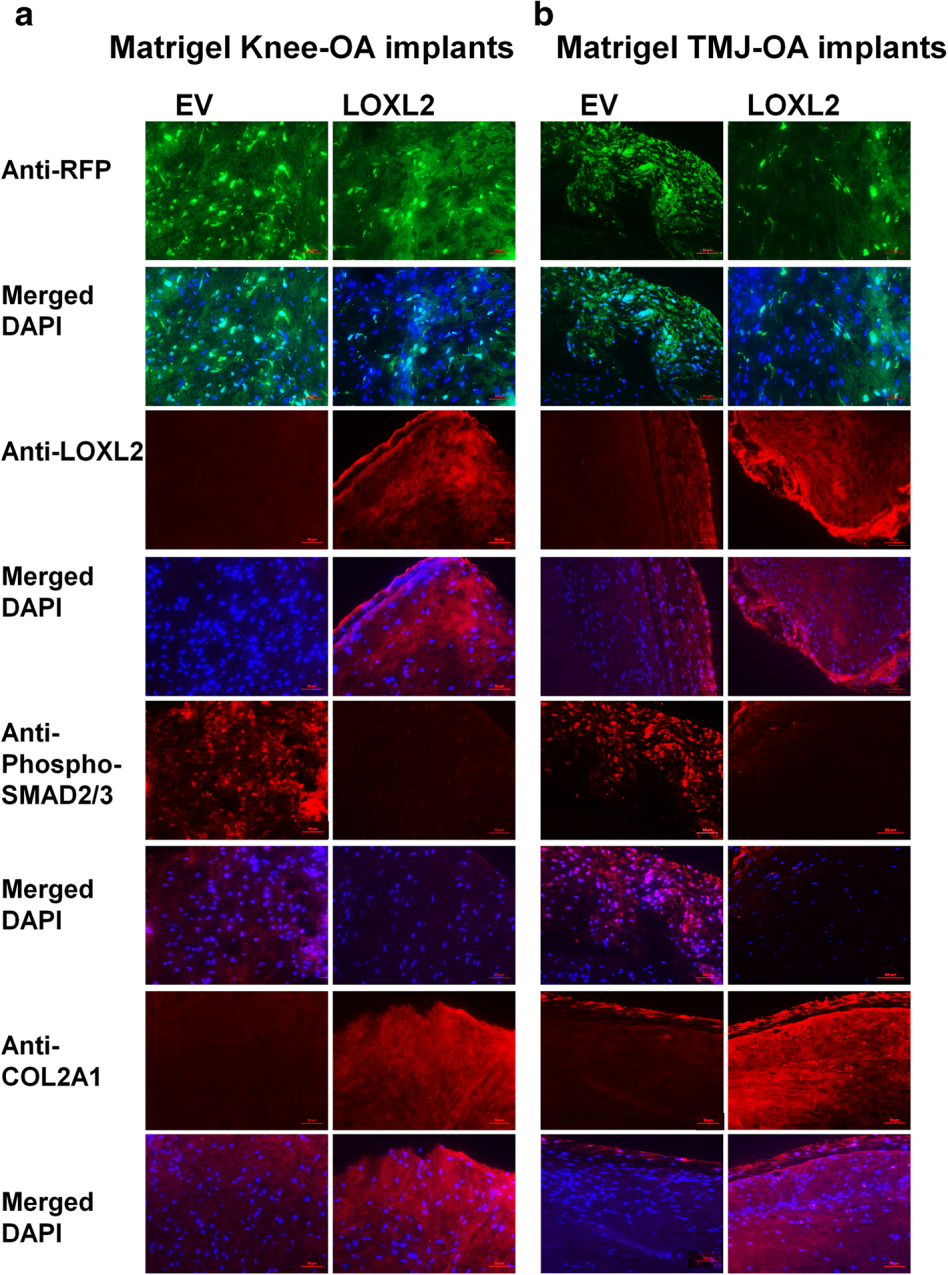
LOXL2 transduction inhibits phospho-SMAD2/3 and increases type II collagen (*COL2A1*). **a** Human knee articular chondrocytes in osteoarthritis (*HAC-OA*)/Matrigel implants and osteoarthritic temporomandibular joint (*TMJ-OA*)/Matrigel implants **b** were stained with antibodies against RFP, LOXL2, phspho-SMAD2/3, or COL2A1 with respective 4′,6-diamidino-2-phenylindole (*DAPI*) staining

To evaluate if LOXL2 can maintain the human cartilage phenotype long term (6 weeks) and induce specific chondrogenic differentiation in vivo, we produced a model of OA using chondrocyte/Matrigel constructs implanted subcutaneously in the backs of nude mice. We used adenoviral delivery of LOXL2 to evaluate the expression in implants of critical genes such as *SOX9*, *ACAN*, and *COL2A1*, which are involved in chondrogenesis. This model is an effective alternative to the human-cell-based models used previously [26–28]: it replicates the 3-dimensional cartilage environment in vivo, uses specific growth factors that preserve the differentiated phenotype for cartilage regeneration [32], and prevents the tissues from becoming fibrotic after 6–9 weeks [33] and myogenic [34]. Weekly injections of Ad-RFP-EV or Ad-RFP-LOXL2 near the chondrocyte-Matrigel implants were performed for up to 6 weeks. IVIS imaging confirmed successful transduction by Ad-RFP-EV or Ad-RFP-LOXL2 (Fig. 6a). After 6 weeks, histological sections of the retrieved implants stained with Safranin O/Fast Green and Alcian blue showed that LOXL2 transduction increased proteoglycan deposition and cartilage formation. However, implants after transduction with Ad-RFP-EV did not show Safranin O or Alcian blue staining, consistent with loss of chondrogenic phenotype and proteoglycan deposition (Fig. 6b). Masson’s trichrome staining was reduced in LOXL2-transduced implants, showing that LOXL2 promotes specific chondrogenic changes and these implants lacked mineralization and fibrosis. In contrast, implants transduced with EV appeared fibrous (Fig. 6b). Molecular analysis of implants by RT-qPCR showed that LOXL2 treatment increased the levels of LOXL2, SOX9, COL2A1, and ACAN mRNA compared to control, whereas MMP13 and ADAMTS5 mRNA levels were not affected significantly in HAC-OA implants (Fig. 6c) and TMJ-OA implants (Fig. 6d). Finally, we evaluated LOXL2 and SOX9 by immunofluorescence staining in HAC-OA and TMJ-OA implant tissue sections. LOXL2 co-localized with nuclear SOX9 (Fig. 7). Adv-RFP-LOXL2 transduction promoted SOX9 gene expression, which was not detected in Ad-RFP-EV-transduced implants, suggesting that LOXL2 maintains the chondrogenic phenotype via SOX9. However, a co-immunoprecipitation assay did not show direct interaction of LOXL2 with SOX9 (Additional file 1: Figure S1). Finally, forced LOXL2 expression resulted in downregulation of phospho-SMAD2/3 and upregulation of COL2A1 compared to Ad-RFP-EV-transduced HAC-OA (Fig. 8a) and TMJ-OA implants (Fig. 8b), which showed lower expression of COL2A1. Thus, LOXL2 transduction may be critical for expression of cartilage-specific matrix genes such as *COL2A1* (Fig. 8a, b). Taken together, our results show that LOXL2 induces an anabolic response in HAC-OA and TMJ-OA chondrocytes in vivo and maintains the chondrogenic phenotype, indicating its essential involvement in cartilage formation and maintenance.

## Discussion

Pro-inflammatory cytokines such as TNF-α and IL-1β act as catabolic factors, promoting synovitis and altering chondrocyte differentiation, function, and viability [35–37]. We showed that LOXL2 attenuated the signaling pathways induced by TGF-β1, TNF-α, and IL-1β. In chondrocytes, IL-1β and TNF-α activate the canonical NF-κB pathway, leading to catabolic and inflammatory responses [38, 39], whereas non-canonical NF-kB signaling promotes chondrocyte hypertrophy [40, 41]. Recent studies showed that TGF-β1 expression in subchondral bone induces OA [42], and TGF-β1 inhibitors are chondroprotective in knee-OA and TMJ-OA in animal models [42–44]. We found that LOXL2 inhibits IL-1β-induced phosphorylation of the NF-κB/p65 subunit, which permits translocation of the p65/p50 complex to the nucleus, and TGF-β1-induced ERK1/2 phosphorylation, which is required for many anabolic responses in cartilage. LOX can directly interact with TGF-β1 and inhibit TGF-β1 activity [45]. Thus, LOXL2 could interact with TGF-β1 to negatively regulate its activity, or influence TNF-α and IL-1β-induced signaling pathways. LOXL2 could also induce amine oxidation at TGF-β1 receptor, inhibiting its activity, or attenuate downstream inflammatory signaling pathways NF-kB and ERK1/2. Indeed, LOXL2 overexpression reduces the expression of the TGF-β-responsive gene PAI1 and negatively regulates TGF-β1-responsive gene sets and SMAD 2/3 phosphorylation. We conclude that LOXL2 could inhibit TGF-β1 signaling, though, alternatively, LOXL2 could be a downstream target of TGF-β1 and act through feedback inhibition of TGF-β1-induced signaling.

LOXL2 is a naturally occurring enzyme and could have potential application in anabolic therapies. The ECM regulates diverse cellular functions, including proliferation, migration, and differentiation, and ECM remodeling is crucial for the development of normal organs. In contrast, dysregulation of the ECM increases LOXL2 expression and contributes to several pathological conditions such as fibrosis and invasive cancer [46–48]. LOXL2 is increased in cancer and fibrosis as a consequence of aberrant signaling pathways and inflammatory mediators leading to remodeling of ECM [49]. Although LOXL2 is present in the damaged regions, the concentration of LOXL2 may be overcome by catabolic mediators such as IL-1β and TNF-α present in degenerative cartilage, which prevent anabolic effects induced by LOXL2 during late stages of OA. These possibilities are under investigation in mouse models to evaluate LOXL2 as a potential therapeutic agent by the early vs. delayed administration, frequency, and dose of application. Current approaches in the development of disease-modifying OA drugs are to: (1) inhibit the ECM-degrading enzymes to prevent erosion of cartilage, (2) inhibit catabolic pro-inflammatory cytokines to protect chondrocytes against stress and inflammatory insults, and (3) develop anabolic agents to promote chondrocyte proliferation and selective differentiation [50]. Unlike other agents that affect one or two targets, our preliminary studies show that LOXL2 could have direct and indirect effects on all of these processes in OA as a potential pro-anabolic and anti-catabolic factor.

Long-term in vivo implanted human chondrocytes lose their chondrogenic phenotype and differentiation potential or become fibrous in the absence of specific stimulating anabolic factors or scaffolds [32, 33]. Our microarray data from both in vitro and in vivo experiments demonstrate that LOXL2 expression is critical for expression of chondrocyte-specific genes such as *SOX9*, *ACAN*, and *COL2A1*. Thus, LOXL2 could have a specific function in maintaining the chondrogenic phenotype. This is also supported by earlier studies showing that LOXL2 knockdown in chondrocytes in vitro inhibits expression of these genes and proteoglycan deposition [6].

We investigated both TMJ and knee joints affected by OA. The TMJ differs in structure compared to other joints [51], with a secondary cartilage distinct from the articular cartilage of limbs and cartilage in the cranial base. However, despite structural and functional differences in the cartilage between the knee joint and TMJ, our findings show that LOXL2 could have roles in the pathophysiology of OA in both types of joint.

Further, we show that LOXL2 co-expresses with SOX9 in the nuclei of both knee OA and TMJ OA chondrocytes in Matrigel implants in nude mice. In other cell types, LOXL2 can localize to the nucleus, perinuclear region, and cytoplasm [52, 53]. LOXL2 interacts physically and functionally with SNAIL to attenuate GSK3β-dependent SNAIL degradation [54] and to downregulate E-cadherin expression [55, 56]. Cytoplasmic/perinuclear localization is associated with distant metastasis of breast cancer cell line [57], while nuclear LOXL2 has a role in epithelial-to-mesenchymal transition [58]. Recent findings also suggest a critical intracellular role for LOXL2 in transcriptional regulation and epigenetic changes [52, 53]. Thus, nuclear LOXL2 could co-localize with SOX9 to cooperatively regulate gene expression. A recent study showed that LOXL2 induces epigenetic changes during neural progenitor differentiation [53]. LOXL2 could, therefore, regulate transcription factors such as SOX9 through direct interactions or indirectly through epigenetic changes. Future investigations of LOXL2-induced epigenetic modifications through enzymatic amine oxidation and other novel mechanisms in OA chondrocytes will provide an additional foundation for understanding its role in OA pathogenesis and for the development of disease-modifying OA drugs.

## Conclusions

**Fig. 9:**
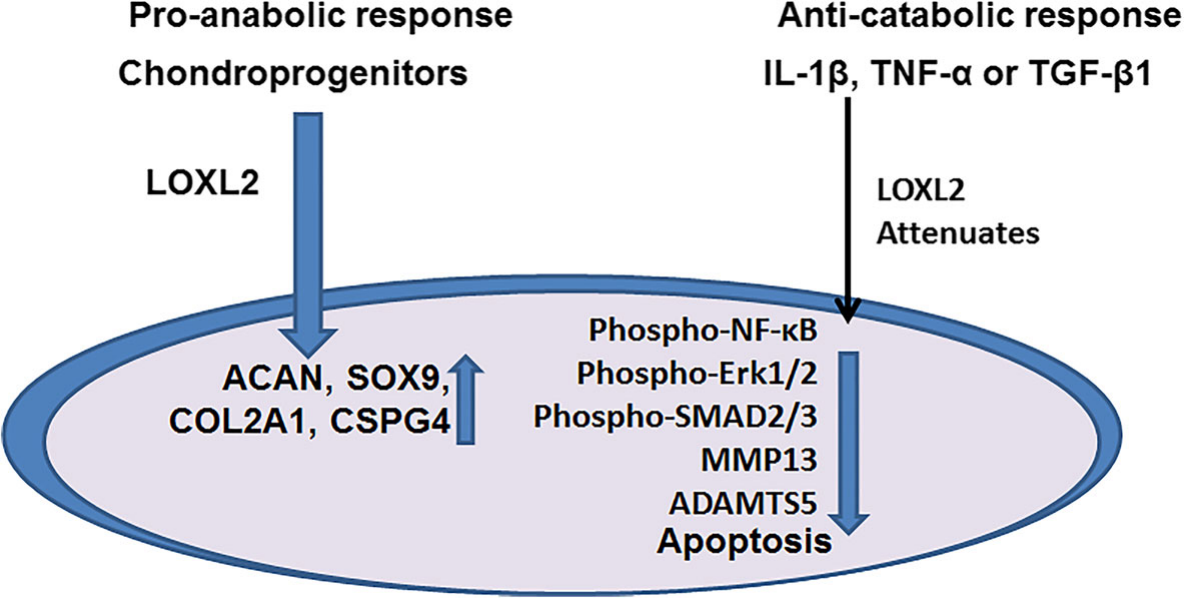
Potential mechanism of LOXL2-induced anabolic effects in osteoarthritis. *TGF* transforming growth factor, ACAN aggrecan, *COL2A1* collagen type II alpha 1, *SOX9* sex determining region Y-box containing gene 9

This is the first study to suggest a potential role for LOXL2 in the pathophysiology of OA (Fig. 9). Our hypothesis that LOXL2 acts as a specific anabolic factor in chondrocytes is supported by data showing that: (1) LOXL2 gene expression is regulated by OA-related mediators; (2) LOXL2 overexpression in chondrocytes inhibits apoptosis induced by TNF-α or IL-1β; (3) LOXL2 overexpression increases SOX9, COL2A1, and ACAN mRNA levels, which are related to the differentiated chondrocyte phenotype, without increasing MMP13 or ADAMTS5; (4) LOXL2 promotes a differentiated phenotype, unlike the dual global effects seen in TGF-β1 [42, 44] and (5) LOXL2 promotes a differentiated phenotype and maintains the chondrogenic lineage in vivo. Taken together, LOXL2 promotes anabolic responses, chondrocyte differentiation, and ECM production. We are evaluating the role of LOXL2 in mouse OA models, which will provide a basic understanding of LOXL2 function for future clinical applications.

## Additional file

Additional file 1: Figure S1. LOXL2 pull-down assay: HAC-OA cells were transduced with Adv-EV or Adv-LOXL2 vector for overnight. The next day, transduced cells were replenished with fresh chondrocyte growth media and cultured for 24 h, and extracted into non-denaturing cell lysis RIPA buffer. The cell lysates were incubated with cobalt chelate NT beads overnight at 4 °C to pull down LOXL2. The beads were washed and eluted according to kit instructions (Pull-Down PolyHis Protein: Protein Interaction Kit Thermo Scientific, Waltham, MA, USA). Eluted samples were analyzed by western blotting on denaturing SDS-PAGE probed with an LOXL2 (Genetex) or SOX9 (Abcam) antibody, and the input (5% aliquots) of initial extracts taken before the immunoprecipitation was analyzed on the same gels. The figure shows pull-down of LOXL2 in IP analysis; however, co-IP with SOX9 does not show any corresponding band. (TIF 50990 kb)

## Abbreviations

ACAN: Aggrecan
Ad-RFP-LOXL2: Adenoviruses for transient expression of LOXL2
BMP: Bone morphogenetic protein
CMV: Cytomegalovirus
COL2A1: Collagen type II alpha 1
CSPG: Chondroitin sulfate proteoglycan
DAPI: 4′,6-Diamidino-2-phenylindole
ECM: Extracellular matrix
ERK: Extracellular signal-related kinase
EV: Empty vector
FBS: Fetal bovine serum
FDR: False discovery rate
GSEA: Gene set enrichment analysis
HAC: Human articular chondrocytes
IL: Interleukin
IRB: Institutional Review Board
IVIS: In vivo imaging systems
LOXL2: Lysyl oxidase like-2
MAP: Mitogen-activated protein
MOI: multiplicity of infection
NES: Normalized enrichment score
Nom *p*-value: Nominal *p*-value
OA: Osteoarthritis
OARSI: Osteoarthritis Research Society International
PBS: Phosphate-buffered saline
RT-qPCR: Quantitative real-time PCR
TGF: Transforming growth factor
SOX9: Sex determining region Y-box containing gene 9
TMJ: Temporomandibular joints
TNF: Tumor necrosis factor
