# Supplementary figures and images for: Anabolic role of lysyl oxidase like-2 in cartilage of knee and temporomandibular joints with osteoarthritis
Source: Arthritis Res Ther. 2017 Aug 2;19:179. doi: 10.1186/s13075-017-1388-8 (PMC5540418; doi:10.1186/s13075-017-1388-8)

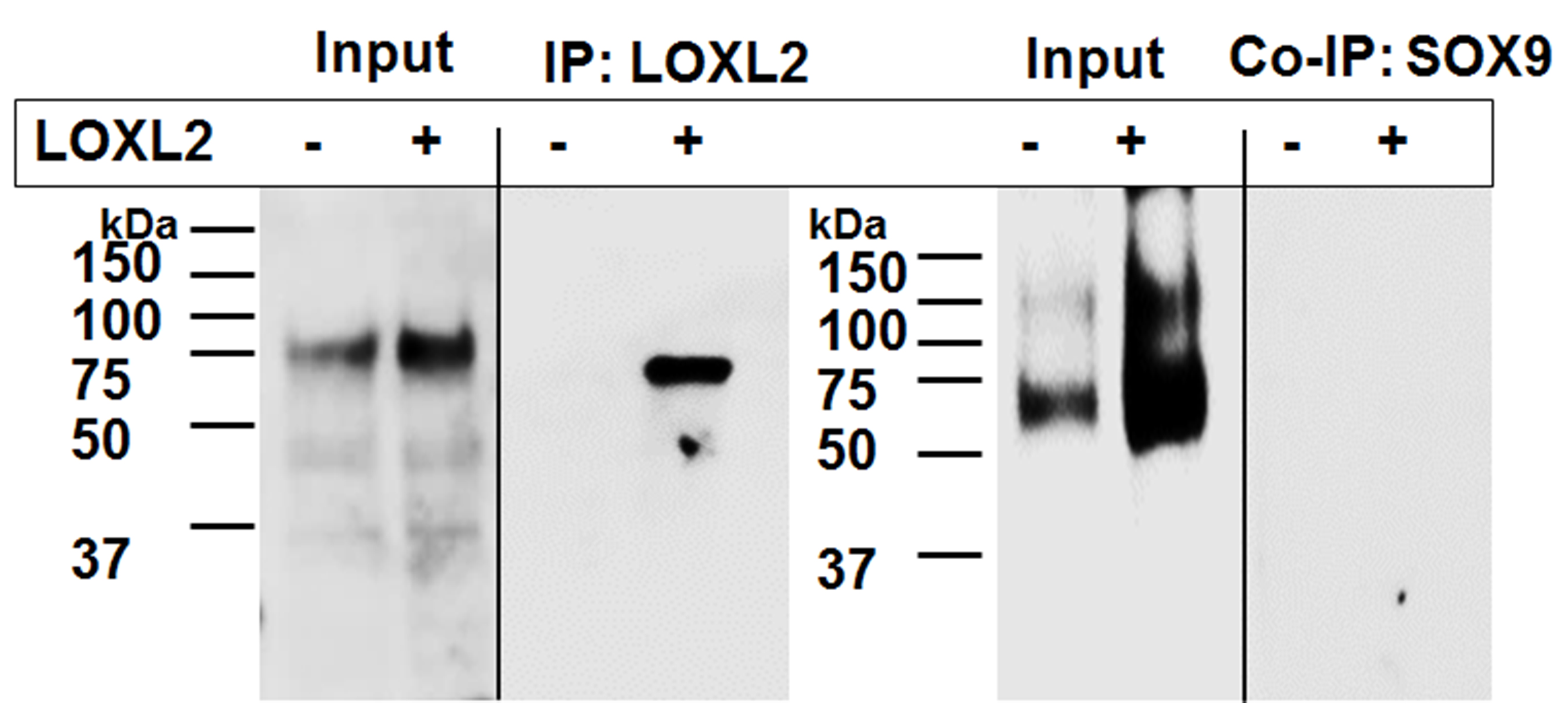

Supplement: Additional file 1: Figure S1. — LOXL2 pull-down assay: HAC-OA cells were transduced with Adv-EV or Adv-LOXL2 vector for overnight. The next day, transduced cells were replenished with fresh chondrocyte growth media and cultured for 24 h, and extracted into non-denaturing cell lysis RIPA buffer. The cell lysates were incubated with cobalt chelate NT beads overnight at 4 °C to pull down LOXL2. The beads were washed and eluted according to kit instructions (Pull-Down PolyHis Protein: Protein Interaction Kit Thermo Scientific, Waltham, MA, USA). Eluted samples were analyzed by western blotting on denaturing SDS-PAGE probed with an LOXL2 (Genetex) or SOX9 (Abcam) antibody, and the input (5% aliquots) of initial extracts taken before the immunoprecipitation was analyzed on the same gels. The figure shows pull-down of LOXL2 in IP analysis; however, co-IP with SOX9 does not show any corresponding band. (TIF 50990 kb) [file 13075_2017_1388_MOESM1_ESM.tif]
